# Kaposi Sarcoma in Afghanistan: A Case Series from a Tertiary Referral Center

**DOI:** 10.3390/dermatopathology9030030

**Published:** 2022-07-15

**Authors:** Alyssa D. Higgins, Richard J. Dunn, Omer Malikzai, Mirwais Ahmadzai, Jerad M. Gardner, Benjamin K. Stoff, Josette R. McMichael

**Affiliations:** 1 Department of Pathology, University of Arkansas for Medical Sciences, 4301 West Markham St., #517, Little Rock, AR 72205, USA; dunrj307@gmail.com; 2 Department of Pathology, CURE Hospital of Kabul, Kabul, Afghanistan; headpathology@beteamintl.org (O.M.); mirwaisahmadzai81@gmail.com (M.A.); 3 Department of Laboratory Medicine, Geisinger Medical Center, Danville, PA 17822, USA; jmgardnermd@gmail.com; 4 Department of Dermatology, Emory University School of Medicine, Atlanta, GA 30322, USA; bstoff@emory.edu (B.K.S.); jmcmichael@emory.edu (J.R.M.)

**Keywords:** Kaposi sarcoma, Afghanistan, human herpesvirus 8 (HHV-8), pathology, dermatopathology, dermatology, telepathology

## Abstract

Kaposi sarcoma is a vascular endothelial neoplasm caused by human herpesvirus 8. Although it is a well-studied disease, little is known about the specific characteristics or epidemiology of Kaposi sarcoma in Afghanistan. The data consist primarily of anecdotal reports and epidemiological studies extrapolated from neighboring countries. In this case series, we summarize existing data about Kaposi sarcoma in Afghanistan and present seven histologically confirmed cases with associated clinical features to shed light on the characteristics of Kaposi sarcoma in this unique geographic setting.

## 1. Introduction

Kaposi sarcoma (KS) was first described by Dr. Moritz Kaposi in 1872 as a multifocal, pigmented sarcoma of the skin, representing an uncommon tumor affecting elderly Mediterranean men. Since that time, KS has garnered attention in association with human immunodeficiency virus (HIV) as an acquired immunodeficiency syndrome (AIDS)-defining neoplasm of endothelial cells, caused by human herpesvirus 8 (HHV-8) [1,2]. As of today, four primary epidemiological forms of KS have been identified—each with defined clinical presentations, risk factors, and prognosis. These forms include the classic, African (endemic), iatrogenic, and AIDS-associated forms of KS [1,2]. With the clinical course ranging from indolent disease to extensive visceral involvement, accurate diagnosis and management can be challenging. There is no cure for KS, and treatment is based on symptom control, disease progression, and cosmesis [1,3]. Optimal control of HIV infection is an integral component of treatment for AIDS-related KS [4,5].

Histologically, KS is characterized by slit-like, vascular spaces which dissect through dermal collagen bundles. The low magnification view of KS is that of a cellular dermal nodule with possible varying degrees of overlying epidermal changes. The dermal proliferation is composed of a spindle-cell proliferation of endothelial cells forming sinuous vascular spaces. These endothelial cells are typically monomorphic with minimal pleomorphism. The presence of the promontory sign may be helpful; however, this finding is not specific to KS. Extravasated red blood cells (RBCs) and hemosiderin deposition are frequent findings [1,2]. The diagnosis of KS can be confirmed by HHV-8 immunostaining, which classically has a speckled/granular pattern of nuclear staining [1].

HHV-8 immunostaining is performed on formalin-fixed, paraffin-embedded tissue using monoclonal antibodies to viral antigens [6]. The pattern and intensity of HHV-8 immunoreactivity may be quite variable from lesion to lesion. Studies have demonstrated that the pattern and intensity of HHV-8 staining is unrelated to patient age, gender, tumor burden, recurrence, location, or epidemiologic form [7,8]. When considering the overall staining pattern of HHV-8, any strong, nuclear positivity of the lesional cells (spindled or endothelial) should be considered positive and supportive of a diagnosis of KS [8].

KS is a well-studied disease, and much data have been reported regarding presentations in many parts of the world [1,2,3,4,5]. However, little is known about the specific characteristics or epidemiology of KS in Afghanistan. Data consist primarily of anecdotal reports and epidemiological studies extrapolated from neighboring countries [3,9,10]. More data are necessary to help guide clinical diagnosis and allocate resources for treatment and improved patient outcomes. In this case series, we summarize existing data about KS in Afghanistan and report seven histologically confirmed cases of KS, alongside the associated clinical features, to contribute to this ongoing effort.

## 2. Case Reports

The cases presented here were collected as part of a collaborative teledermatopathology effort between the Maiwand Teaching Hospital Dermatology Department in Kabul, Afghanistan; CURE International Hospital in Kabul, Afghanistan; Emory University in Atlanta, Georgia; and the University of Arkansas for Medical Sciences in Little Rock, Arkansas. In this model, patients were referred to Maiwand for further workup and treatment. Skin biopsies were then sent to CURE International Hospital in Kabul for pathologic diagnosis. The results were documented, photographed, and forwarded to a dermatopathologist at Emory University, who stained the blocks with HHV-8 and provided interpretation and diagnosis. Additional feedback was provided by a dermatopathologist and soft tissue pathologist at the University of Arkansas for Medical Sciences. This type of collaborative effort has been reported previously with beneficial outcomes [11].

Seven patients were evaluated in the Dermatology Department at Maiwand Teaching Hospital, located in Kabul, Afghanistan. The majority (4/7) were from the remote Northern provinces of Afghanistan, and all presented with violaceous nodules and papules in varying distributions. Only one patient tested HIV-positive. Patient demographics, presentation, and histology are summarized in Table 1. All cases are reviewed as follows:

### 2.1. Case 1

**Figure 1:**
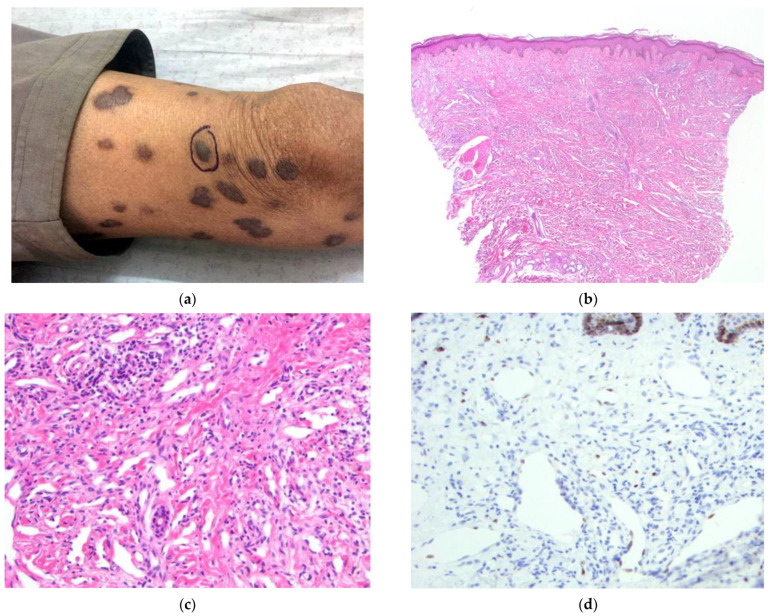
(**a**–**d**) Patient #1. Image (**a**) Multiple, violaceous patches and nodules of the lower extremities. Biopsy site shown. Image (**b**) H&E 40×. Dermal infiltration by slit-like spaces. Image (**c**) H&E 200×. Vascular spaces lined by bland, spindled endothelial cells. No significant RBC extravasation is identified. Image (**d**) IHC HHV-8 200×. Focal nuclear positivity of spindled endothelial cells within the dermis.

The patient (#1) (Figure 1) is a 63-year-old male from Kabul who presented with a five-year history of generalized and increasing violaceous papules and nodules of the trunk and extremities (Figure 1a). Physical examination also revealed a lesion of the soft palate and facial hyperpigmentation. HIV testing was negative. The clinical presentation strongly favored a diagnosis of KS. Additional diagnostic considerations included leukemia/lymphoma, cutaneous metastasis, angiosarcoma, or deep fungal infection.

A 4 mm punch biopsy was obtained from a well-developed nodule of the left leg and showed infiltration of the dermis by slit-like vascular spaces lined by spindled endothelial cells. There was no extravasation of RBCs or significant atypia of the endothelium (Figure 1b,c). A diagnosis of KS was made. Immunohistochemical staining for HHV-8 confirmed the diagnosis of KS (Figure 1d).

### 2.2. Case 2

**Figure 2:**
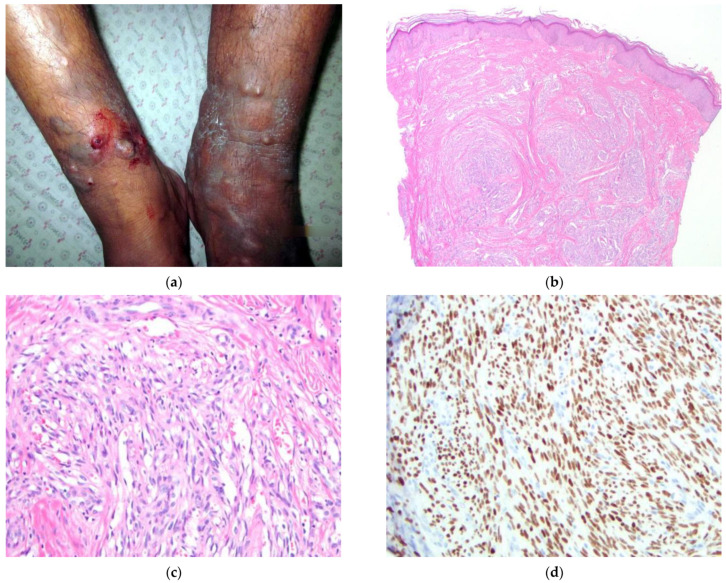
(**a**–**d**) Patient #2. Image (**a**) Multiple, violaceous patches, nodules, and papules of bilateral lower extremities (left > right) with mild lymphedema of left leg. Image (**b**) H&E 40×. Multiple nodules within the deep dermis. Image (**c**) H&E 200×. Spindled cell proliferation forming slit-like spaces. No significant RBC extravasation is identified. Image (**d**) IHC HHV-8 200×. Strong and diffuse nuclear staining of spindled cells within the dermis.

The patient (#2) (Figure 2) is a 60-year-old male from a remote province who presented with a three-year history of increasing edema and painful, pruritic nodules of the bilateral lower extremities. He was previously evaluated with a biopsy of a non-nodular area which was histologically compatible with acroangiodermatitis (pseudo-KS; a manifestation of advanced venous stasis). Due to the persistent and pruritic nature of the lesions, this patient sought a second opinion. Physical examination demonstrated violaceous nodules of the bilateral lower extremities (Figure 2a) with scattered, similar lesions of the bilateral distal upper extremities. Many well-developed lesions demonstrated central ulcerations and erosions. HIV testing was negative. The clinical presentation and previous biopsy results favored a diagnosis of acroangiodermatitis. Additional diagnostic considerations included KS and angiosarcoma.

A 4 mm punch biopsy was obtained from a nodule of the right leg and showed fascicles of spindle cells with slit-like spaces. Erythrocytes were present in vessels, but without much extravasation. There was an element of background stasis-change in the superficial dermis (Figure 2b,c). The differential included KS and acroangiodermatitis. The presence of a spindle cell population and interstitial dermal growth, coupled with the clinical history of lesions affecting the upper extremities and the lack of clinical findings supportive of advanced lymphovascular stasis, favored a diagnosis of KS. Immunohistochemical staining for HHV-8 confirmed the diagnosis of KS (Figure 2d).

### 2.3. Case 3

**Figure 3:**
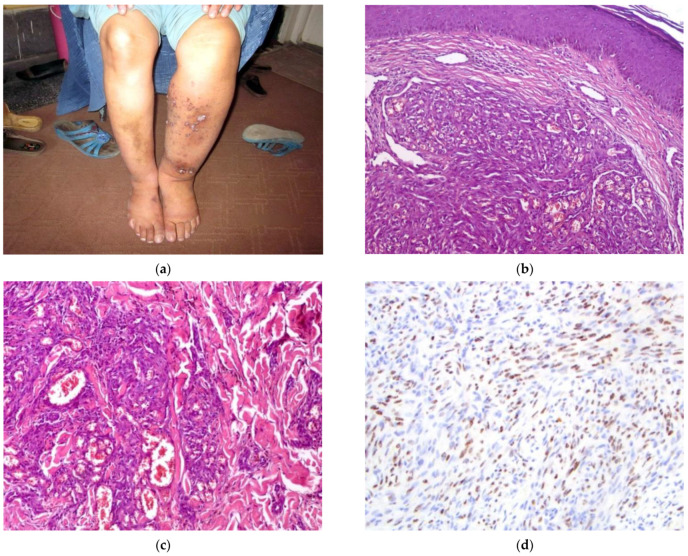
(**a**–**d**) Patient #3. Image (**a**) Multiple violaceous patches, nodules, and papules in the setting of unilateral lymphedema in the left lower extremity. Image (**b**) H&E 40×. Nodules of uniform spindle cells in a fascicular pattern. Image (**c**) H&E 100×. Area of nodularity located in deep dermal tissues. No significant RBC extravasation is identified. Image (**d**) IHC HHV-8 200×. Strong and diffuse nuclear staining of spindled cells within the dermis.

The patient (#3) (Figure 3) is a 45-year-old female from a remote province with a four-year history of unilateral left lower extremity edema, followed by a seven-month history of increasing violaceous papules and nodules of the same extremity (Figure 3a). These lesions were pruritic but otherwise asymptomatic. Physical examination revealed no additional lesions. HIV testing was negative. The clinical presentation initially favored angiosarcoma with other diagnostic considerations, including deep fungal infection, KS, or bacillary angiomatosis.

A 4 mm punch biopsy of the left lower extremity was obtained and showed dermal nodules of uniform, spindled cells with slit-like vascular spaces. Some ectatic vascular channels are identified, along with eccrine coil involvement. Few extravasated RBCs are identified (Figure 3b,c). A diagnosis of KS was favored. Immunohistochemical staining for HHV-8 confirmed the diagnosis of KS (Figure 3d).

### 2.4. Case 4

**Figure 4:**
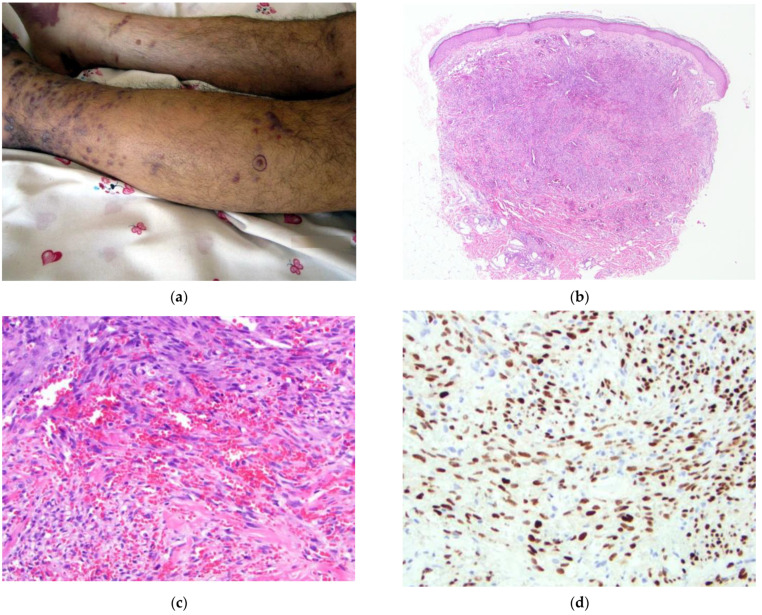
(**a**–**d**) Patient #4. Image (**a**) Multiple violaceous patches, nodules, and papules of the bilateral lower extremities with more significant involvement of the left leg. Biopsy site shown. Image (**b**) H&E 40×. Dermal-based nodularity. Image (**c**) H&E 200×. Spindled cell proliferation forming slit-like spaces. RBC extravasation is present. Image (**d**) IHC HHV-8 200×. Strong and diffuse nuclear staining of spindled cells within the dermis.

The patient (#4) (Figure 4) is a 61-year-old male from a rural area in a remote province who presents with a one-year history of violaceous papules, nodules, and patches of the bilateral lower legs (left > right) (Figure 4a) and similar lesions of the dorsal aspects of the hands. Physical examination revealed significant lymphedema of the left lower extremity. HIV testing was negative. The clinical presentation strongly favored a diagnosis of KS. Additional diagnostic considerations included angiosarcoma or metastatic malignancy.

A 4 mm punch biopsy of a representative papule of the left lower leg was obtained and showed orthokeratosis with a dermal nodule composed of vascular channels with a flattened endothelial lining. Endothelial nuclei were bland, and some RBC extravasation was noted (Figure 4b,c). A diagnosis of KS was made. Immunohistochemical staining for HHV-8 confirmed the diagnosis of KS (Figure 4d).

### 2.5. Case 5

**Figure 5:**
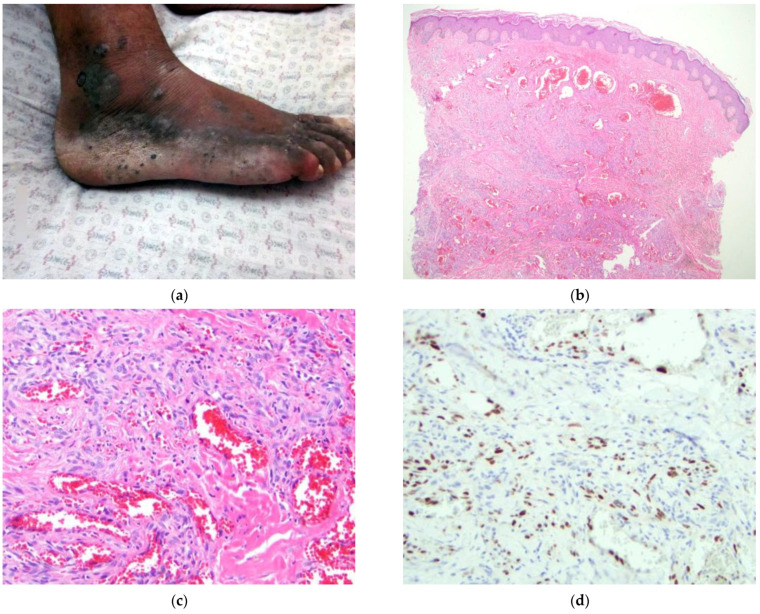
(**a**–**d**) Patient #5. Image (**a**) Multiple purple/black nodules and papules on the right lower extremity. Biopsy site shown. Image (**b**) H&E 40×. Dilated vascular spaces of varying caliber within the superficial and deep dermis. Image (**c**) H&E 200×. Bland nuclei with mild atypia forming slit-like vascular spaces. Image (**d**) IHC HHV-8 200×. Scattered nuclear staining of spindled cells within the dermis.

The patient (#5) (Figure 5) is a 68-year-old male from a rural area in a remote province who presents with a two-year history of painful, enlarging, violaceous and black nodules and papules of the right dorsal and plantar foot with a few scattered similar lesions of the ankle and anterior lower extremity (Figure 5a). Physical examination revealed additional, violaceous, non-blanching macules and small patches of the left plantar foot, of which the patient was previously unaware. HIV testing was negative. The clinical presentation strongly favored a diagnosis of KS. Additional diagnostic considerations included deep fungal infection, angiosarcoma, and less likely, melanoma.

A 4 mm punch biopsy of the right ankle was obtained and showed a dermal proliferation of small and large blood vessels with some extravasation of erythrocytes. There were some spindle cells that were not overtly atypical (Figure 5b,c). KS was favored. Immunohistochemical staining for HHV-8 confirmed the diagnosis of KS (Figure 5d).

### 2.6. Case 6

**Figure 6:**
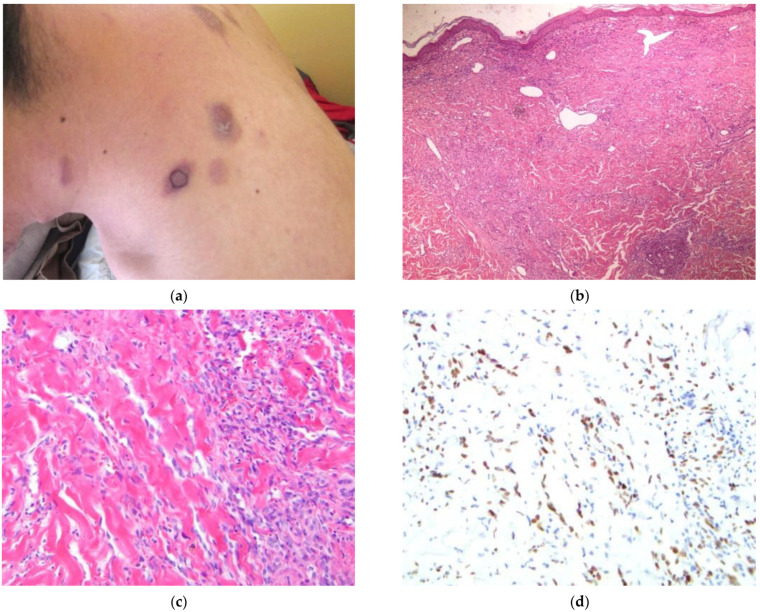
(**a**–**d**) Patient #6. Image (**a**) Violaceous patches and nodules on the posterior neck and trunk. Biopsy site shown. Image (**b**) H&E 40×. Spindled cells with collagen infiltration, slit-like spaces, and extravasated RBCs. Image (**c**) H&E 200×. Proliferation of spindled cells with slit-like spaces infiltrate into adjacent collagen. Image (**d**) IHC HHV-8 200×. Scattered nuclear staining of spindled cells within the dermis.

The patient (#6) (Figure 6) is a 27-year-old male from Kabul with a history of intravenous heroin use who presented with a four-month history of increasing, indurated, violaceous nodules scattered on the face, trunk, and extremities (Figure 6a). Physical examination also revealed facial edema and lesions of the oral mucosa. HIV testing was positive. The clinical presentation strongly favored a diagnosis of KS. Additional diagnostic considerations included mycosis fungoides, other cutaneous T-cell lymphomas, or metastatic disease.

A 5 mm punch biopsy from the center of an indurated nodule on the left shoulder showed a proliferation of pleomorphic, plump, spindled cells in the mid-dermis. This lesion demonstrated infiltration of surrounding collagen by slit-like spaces and abundant extravasated RBCs (Figure 6b,c). A diagnosis of KS was made. Immunohistochemical staining for HHV-8 confirmed the diagnosis of KS (Figure 6d).

### 2.7. Case 7

**Figure 7:**
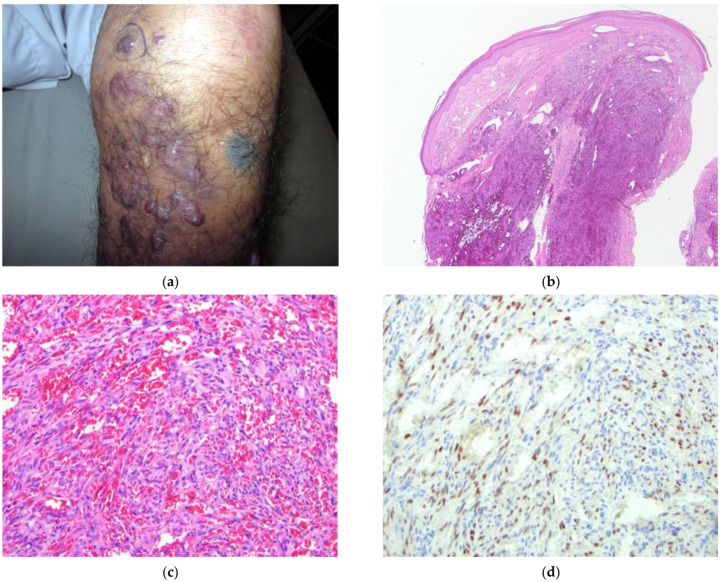
(**a**–**d**) Patient #7. Image (**a**) Lower leg with multiple, confluent violaceous nodules. Area of blue pigmentation/cutaneous discoloration represents tattoo ink. Biopsy site shown. Image (**b**) H&E 40×. Nodular proliferation within the superficial and deep dermis. Image (**c**) H&E 200×. Vascular proliferation with flattened epithelium and extravasated RBCs. Image (**d**) IHC HHV-8 200×. Scattered nuclear staining of spindled cells within the dermis.

The patient (#7) (Figure 7) is an 85-year-old male from Kabul with a one-year history of enlarging, confluent, violaceous plaques and nodules of the bilateral lower legs and feet (Figure 7a). Physical examination revealed small, similar lesions on the forearms and a few small, erythematous macules on the soft palate. Additional blue-black patches were observed on the knees and, by history, were identified as tattoos placed as a traditional therapy to remedy these lesions. HIV testing was negative. The clinical presentation strongly favored a diagnosis of KS. Additional diagnostic considerations included angiosarcoma, metastatic carcinoma, lymphoma, and changes compatible with chronic lymphedema.

A 4 mm skin punch biopsy of a nodule on the right lower leg was obtained and revealed closely packed blood vessels lined by spindled endothelial linings with some extravasation of erythrocytes within the dermis (Figure 7b,c). KS was strongly considered, as was spindle cell hemangioma. Clinical correlation was recommended. Immunohistochemical staining for HHV-8 confirmed the diagnosis of KS (Figure 7d).

## 3. Discussion

We found a predominance of KS in men, primarily from the Northern provinces of Afghanistan. The patients presented with characteristic clinical features. Lesions tended to be multifocal (5/7, 71%), with a predilection for the lower extremities (7/7, 100%), upper extremities (5/7, 71%), and oral mucosa (3/7, 43%). KS lesions were also identified involving the trunk (2/7, 29%). The majority of patients were older men; one middle-aged woman; and one younger, adult male (mean age 58.43; SD 16.88). Only one of the seven patients tested positive for HIV. This individual’s HIV status was previously unknown, and the clinical suspicion of KS prompted HIV screening.

Since the majority of these cases (6/7) are present in AIDS-negative individuals and lack clinical features identified of other subtypes of KS, they are best classified as examples of classic KS. This finding is not unexpected, as classic KS, although rare, is most common in individuals of Mediterranean or Middle Eastern descent [5]. A possible exception is case 1. Despite an initial negative HIV test, we suspected a false negative and sent him for repeat testing. Unfortunately, he was then lost to follow-up. The limited resources and remote origin of many of these patients greatly impeded the follow-up of many. To our knowledge, none of the patients had a concurrent or subsequent additional neoplasm.

Many of the traditional histologic features of KS were represented. All cases demonstrated classic, endothelial-lined, slit-like spaces. Five of the seven cases exhibited some degree of RBC extravasation—three of which were qualified with descriptors such as “few” or “some”. Two cases did not display any appreciable extravasated RBCs. Features such as eccrine coil involvement, a well-developed promontory sign, and a fascicular endothelial growth pattern were all variably represented. In all cases, a diagnosis of KS was confirmed by demonstrating HHV-8 expression by immunohistochemistry. The patient characteristics are summarized in Table 1.

Based on data from the International Agency for Research on Cancer (IARC), KS has an incidence rate of 0.01/100,000 people per year in Afghanistan. However, this number is derived from averages in surrounding countries, such as Iran and India [2,9,10,12]. A thorough search of the existing literature reveals one anecdotal report indicating that the predominant clinical variant found in Afghanistan is classic KS, which our findings generally support [3,9]. One case in our series is AIDS-associated, which, to our knowledge, has not been previously reported in Afghanistan. Despite our findings, much remains unknown surrounding KS in Afghanistan. First, the seroprevalence of HHV-8 in Afghanistan has not been defined [3,9,10,11]. Additionally, data regarding HIV rates in Afghanistan are also sparse—data available on the HIV and AIDS Data Hub for Asia-Pacific (APHUB) record 791 individuals on antiretroviral therapy, but they do not speak to the incidence, prevalence, or survival of these people [13].

A diagnostic challenge for this population is that general healthcare services and pathology services are severely limited or non-existent in many provinces in Afghanistan. Individuals tend to present in more advanced stages due to a lack of, or inability to seek out, healthcare. Pathology services are centralized and found in more-urban locations; patients from rural provinces are often unable to obtain a histologic diagnosis until they travel to the capital city of Kabul. Travel to any city, let alone Kabul, may prove an insurmountable task for some individuals due to the remoteness of their provinces. This ultimately results in delayed diagnosis and inappropriate treatments.

Regarding the diagnosis of KS specifically, one of the barriers in pathology is the lack of availability of diagnostic, immunohistochemical stains, such as that for HHV-8. Immunohistochemical stains generally require a higher volume of cases and appropriate quality controls. Therefore, clinical suspicion and hematoxylin- and eosin-based light microscopy are the most valuable tools. Follow-up for these patients brings its own unique challenges, and waiting for the results of HHV-8 stains may be impractical [11,12,13].

Telemedicine and telepathology services are burgeoning healthcare-delivery platforms. These services provide opportunities to expand access to healthcare to underserved populations in remote areas, such as developing countries [14]. Access to specialty services is highly variable throughout the world, with many developing countries having limited or no access to dermatopathologists and advanced laboratory services [3,9,10,11,14]. To address these healthcare disparities, providers have turned to telemedicine, specifically telepathology consultation on diseases requiring clinical-pathologic correlation.

Finally, one patient (#6) was HIV positive with a history of heroin abuse. Little research has been done on illicit drug use in Afghanistan, even though most of the world’s opium originates in the country [15]. Ongoing political unrest, geographical challenges, a rural and mobile population, and cultural norms make evaluation of the true prevalence of illicit drug use challenging [15,16]. As reported by Cottler et al., approximately 5% of the Afghan population either reported or had biological evidence of recent drug use (opium, heroin, or pharmaceutical opioids) [17]. Minimal literature exists examining rates of HIV transmission and illicit drug use within the general population [17,18]. Future investigations into these related topics may one day provide more insight into the prevalence of KS.

Our interest in this topic was driven by the needs of local healthcare providers and the paucity of published data regarding dermatologic diseases in a largely underserved population. To date, data consist mainly of anecdotal reports and epidemiological studies extrapolated from neighboring countries. This case series represents an international collaboration between dermatologists, general pathologists, dermatopathologists, and soft tissue pathologists across multiple countries. The unique opportunities afforded by this collaborative effort have allowed for the accurate diagnosis of what is likely an underreported disease in the Afghan population. This study demonstrates that collaboration can facilitate access to resources, such as immunohistochemical stains, which increases diagnostic accuracy.

Future goals of this project include evaluation of treatment modalities, patient follow-up to assess disease status, and documentation of co-morbidities. This study is limited due to the paucity of cases and the referral bias of the patients. It is unknown whether individuals from represented provinces are more able to make the arduous journey for a more-advanced level of care. Alternatively, it is possible that there is a higher incidence of KS in these provinces. It is likely that these patients represent a small subset of KS cases in Afghanistan.

## 4. Conclusions

This case series contributes valuable information to the body of knowledge for KS in Afghanistan, with the hope that this will increase awareness and, ultimately, improve outcomes for this disease.

## Figures and Tables

**Table 1:** Characteristics of patients with Kaposi sarcoma.

ID #	Age	Sex	Province	HIV Status	Lesion Morphology	Lesion Location	Histologic Features
1	63	Male	Kabul	Negative	Violaceous papules and nodules	Trunk, extremities, soft palate	Slit-like vascular spaces Lined with spindled endothelial cells Negative for RBC extravasation Negative for significant atypia
2	60	Male	Faryab	Negative	Painful, pruritic nodules, some with ulceration	Bilateral lower legs, distal upper extremities	Nodules of spindle cells Vascular proliferation, slit-like spaces Few extravasated RBCs Negative for significant cytologic atypia
3	45	Female	Takhar	Negative	Violaceous, pruritic, painless papules and nodules	Left lower leg	Nodules of uniform spindle cells in a fascicular pattern Slit-like vascular spaces Ectatic vascular channels Eccrine coil involvement Few extravasated RBCs
4	61	Male	Takhar	Negative	Violaceous papules, nodules, and patches	Bilateral lower legs, dorsal hands	Dermal nodule of vascular channels Flattened endothelial lining Bland cytologic features Some RBC extravasation
5	68	Male	Faryab	Negative	Painful nodules and papules	Right dorsal and plantar foot, ankles, and anterior lower leg, left plantar foot	Dermal proliferation of dilated small and large blood vessels Spindle cells Slit-like vascular spaces Extravasated RBCs
6	27	Male	Kabul	Positive	Indurated, violaceous nodules	Face, trunk, extremities, oral mucosa	Mid-dermal pleomorphic, plump, spindled cells Collagen infiltration Slit-like spaces Extravasated RBCs
7	85	Male	Kabul	Negative	Confluent, nodular, violaceous plaques	Bilateral lower legs and feet, bilateral forearms, soft palate	Closely packed dermal spindle cell nodule Flattened endothelial cells Negative for significant cytologic atypia Negative for RBC extravasation

## Data Availability

All generated data is presented within the article.
